# Data Quality Assessment and Multi-Organizational Reporting: Tools to Enhance Network Knowledge

**DOI:** 10.5334/egems.280

**Published:** 2019-03-29

**Authors:** Sanchita Sengupta, Don Bachman, Reesa Laws, Gwyn Saylor, Jenny Staab, Daniel Vaughn, Qing Zhou, Alan Bauck

**Affiliations:** 1 Kaiser Permanente Northwest, Center for Health Research, US

**Keywords:** Virtual Data Warehouse, Multi-organizational Data Quality Assessment, Data Network, Multi-organizational Research

## Abstract

**Objective:**

Multi-organizational research requires a multi-organizational data quality assessment (DQA) process that combines and compares data across participating organizations. We demonstrate how such a DQA approach complements traditional checks of internal reliability and validity by allowing for assessments of data consistency and the evaluation of data patterns in the absence of an external “gold standard.”

**Methods:**

We describe the DQA process employed by the Data Coordinating Center (DCC) for Kaiser Permanente’s (KP) Center for Effectiveness and Safety Research (CESR). We emphasize the CESR DQA reporting system that compares data summaries from the eight KP organizations in a consistent, standardized manner.

**Results:**

We provide examples of multi-organization comparisons from DQA to confirm expectations about different aspects of data quality. These include: 1) comparison of direct data extraction from the electronic health records (EHR) and 2) comparison of non-EHR data from disparate sources.

**Discussion:**

The CESR DCC has developed codes and procedures for efficiently implementing and reporting DQA. The CESR DCC approach is to 1) distribute DQA tools to empower data managers at each organization to assess their data quality at any time, 2) summarize and disseminate findings to address data shortfalls or document idiosyncrasies, and 3) engage data managers and end-users in an exchange of knowledge about the quality and its fitness for use.

**Conclusion:**

The KP CESR DQA model is applicable to networks hoping to improve data quality. The multi-organizational reporting system promotes transparency of DQA, adds to network knowledge about data quality, and informs research.

## Introduction

Continuous maintenance and assessment of data in a distributed network ensures high quality, consistent, and easy to use data [1,2,3,4,5]. In a distributed data network, each participating region collects data from multiple sources and transforms the data to fit the data specifications of a common data model. These networks are important for research as they allow researchers to assemble large populations in which to study uncommon conditions. For Kaiser Permanente’s Center for Effectiveness and Safety Research (CESR), the common data model is the CESR virtual data warehouse (VDW). For this VDW, data is collected from all elements of an integrated health care organization.

One of the complications of assembling such a large data resource is that the information on a single encounter or event may be found in multiple sources in each health system—electronic health records (EHR), claims data, membership data, coverage and benefits, patient registries, disease registries, pharmacy, birth and death certificates, and other internal sources. Each of these sources captures a specific footprint of the patient in the health care system. For example, death data has multiple different sources, both internal and external to the organizations, due to death of members occurring inside or outside facilities. Combining data from all sources is important to get complete death information on all members. Since all available sources are considered for building the VDW death data, issues related to data linkage and completeness are important factors in assuring data quality. Contrary to this, patient reported social history data on tobacco/alcohol/illicit drug use, birth control, and sexual behavior for the VDW are almost exclusively obtained from the EHR in more recent years. For social history, the methods and frequency of data collection through EHR at different organizations is key to understanding the underlying data differences amongst organizations. These are two examples as to why data scientists must conduct frequent data quality assessments and compare data for similarities and differences between organizations to ensure and assess the quality of the data.

Quality assessments are essential for multisite research. Having consistent and standardized data quality assessments (DQA) across all regions allows researchers to generate metadata from each region that can be combined for multi-organization assessment. In a distributed network, transparent and consistent multi-site reporting is a powerful tool to understand data, identify errors, and perform across organization comparisons that helps researchers and data managers learn from the data [6]. This is especially true for cases where known data standards for completeness do not exist, clinical expectations are not defined, or there is a lack of understanding of data provenance (the context under which the data was collected) [7]. Callahan and colleagues [8], for example, compared DQA amongst six different data sharing networks using the harmonized DQA terminology developed by Kahn and colleagues [5]. Both papers emphasize that expectations about data are based on comparisons to local knowledge that is gained from a data source like the VDW and the data generation processes. As such, a clearly articulated and executed DQA is essential for successful multi-site data studies.

To this end, this paper discusses how users can compare data summaries from several different organizations to formulate an implicit standard against which to validate a single organization’s data expectations. We discuss a tool that helps in accumulating local knowledge for enhancing network knowledge. We compare the standardized DQC results across eight independent Kaiser Permanente (KP) organizations with known health care infrastructure. For this discussion, we will use social history data as our focus. Since social history data is obtained from a single source common to all organizations, we would expect to find similar trends in the data. Differences in data might be attributed to the differences in the set-up of the EHR across regions. For example, the health care software version of Kaiser Permanente Washington is different from that of other KP research centers. In contrast, the differences across organizations on the sources of death data might be responsible for the variation in data quality as well as availability which can be identified when the trend data from different organizations are placed together.

The KP CESR DQA process involves using two levels of reporting. The first level improves regional data quality and enhances local knowledge. Each organization runs a common QA program that provides summary statistics on all variables compiled in a report. The data managers update extract, load, and transform (ETL) programs if there are fail messages in the report. The second process improves data quality by identifying data patterns that are inconsistent with cross-organizational distributions. The metadata created during the process is a knowledge repository that is available to all researchers in the network. By describing this process, this paper fills a gap in the literature on the usage of aggregate data summaries generated from a DQA. The CESR DQA model is replicable across organizational networks working with a common data model and helps overcome many of the challenges of working with multi-organization data.

## Methods

### Kaiser Permanente, Center for Effectiveness and Safety Research, Data Coordinating Center

Kaiser Permanente (KP) is an integrated managed care delivery system comprising of a network of eight independent organizations. KP provides cost effective care largely through integration of funding with provision of services. The Center for Effectiveness and Safety Research (CESR) is a national research collaborative comprising KP’s research centers in eight regions: Colorado, Georgia, Hawaii, Mid-Atlantic States (Maryland and Virginia), Northern California, Northwest (Oregon/South Washington), Southern California and Washington. The Data Coordinating Center (DCC) facilitates a standardized distributed data model for research called CESR Virtual Data Warehouse (VDW). The CESR VDW builds upon the Health Care Systems Research Network (HCSRN) VDW [9] and supports its ongoing development.

### CESR VDW Quality Assessment Process

The CESR DCC performs regular QA on the VDW to ensure data is of high quality and meets data specification standards. As in many other distributed data networks, the data owners maintain possession of the individual level data within their organizations and determine the data use and accessibility. All organizations, however, participate in the same DQA process that is common across organizations. Any project or organization-specific research data requirement could be met by supplementing this common baseline QA with project-specific QA.

The CESR DCC conducts QA for one data content area at a time, with each dataset undergoing QA at least once every two years or more often if there is a specification change or data source update. The timing of a specific QA does not depend on data refresh cycle as these are site specific.^1^

**Figure 1:**
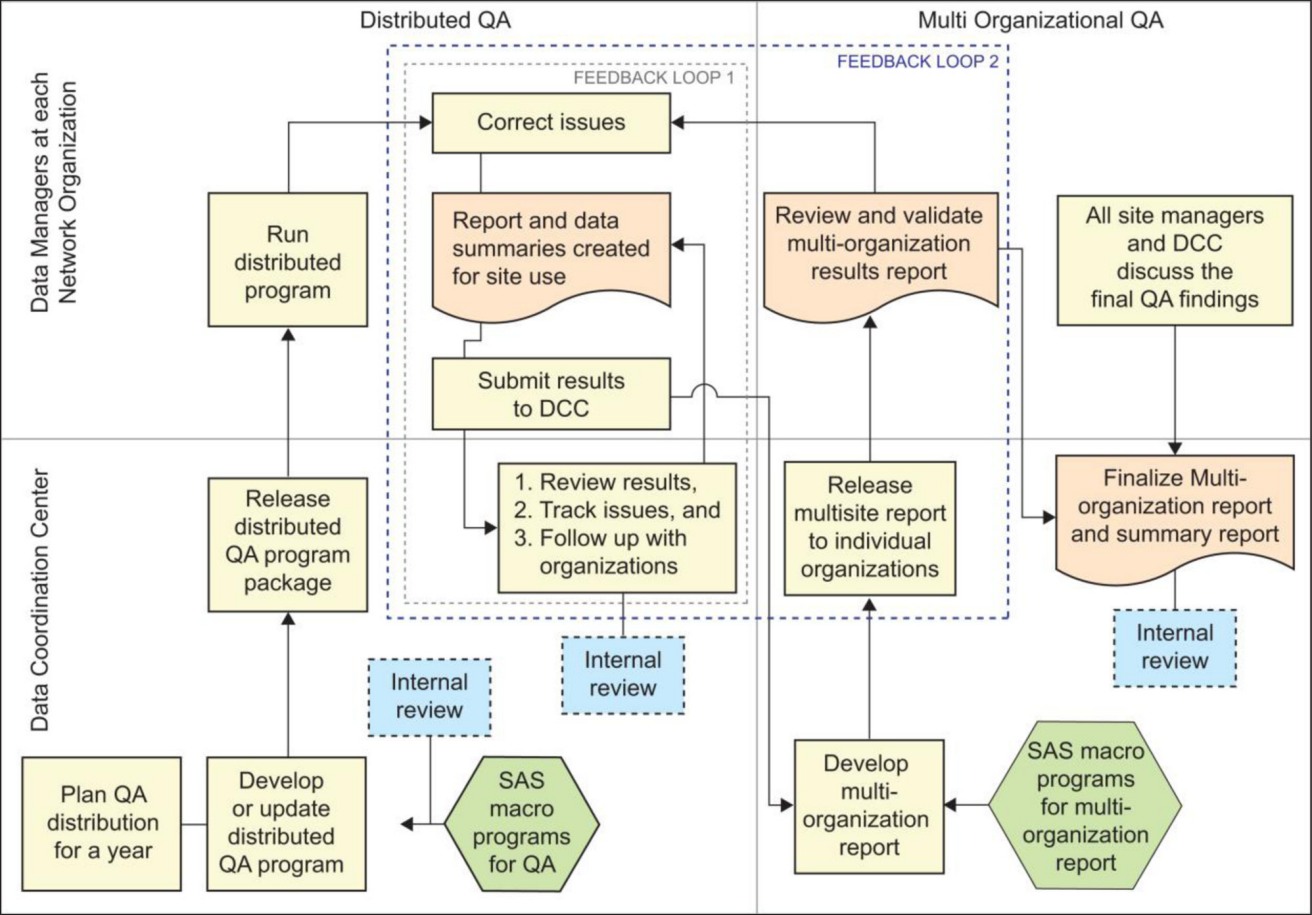
Flowchart on the Distributed and Centralized DQA Processes of CESR DCC.

Figure 1 below shows the workflow between DCC analysts/consultants and the data managers in each organization. The QA process consists of two levels:

1. **Distributed QA:** The DCC updates, tests, and distributes the QA package. Each organization runs the QA package, reviews the results, notes the warning and fails issued in the program run, and corrects the data accordingly. The QA program generates a data outcome file and multiple metadata files that are then sent back to the DCC.
2. **Centralized QA:** The DCC combines QA meta-file results from each organization into one single data structure. They provide feedback to individual organizations after a multi-organization comparison of the metadata. Every time a data update is requested from the DCC, the organizations rerun and resend back the QA program results and the multi-organization report is updated. The DCC then refers to this document to communicate either with individual regions on specific issues or collectively in a meeting on issues that are common across systems.

### CESR VDW Quality Assessment Techniques

The organization-level CESR DQA approach and techniques have been discussed in papers cited above. In the harmonized data quality assessment terminology [8], all DQ checks fall into specific domains such as the following:

1. Value checks (e.g., death date is an invalid value like 13/1/9999)
2. Relational (e.g., do the members in the death file match the members in the demographics file?) and computational (e.g., do members in the death file have death dates before birth dates in the demographics file) conformance checks
3. Completeness (i.e., are we missing death information on any members?) and plausibility (e.g., do events occur after death date?)

The results from all these QA checks are generated in a single report as soon as the site data managers run the DQA.

Aggregate data summaries include counts of missing values for each data field, distribution of data field by population sub-type like gender or age-group, record counts by range of values, record counts by year or as proportion of enrolled members, unique records grouped or sub-grouped by primary keys, and counts of data where foreign keys (usually the unique identifier for patient or encounter) that link with other VDW tables. These data summaries are available from all organizations as metadata from a DQA process and are combined in the summary report. To compare data build and capture over time, for example, CESR DCC uses total annual and monthly counts. These counts are converted to rates/proportions by taking a common denominator of number of enrolled members [3] during the time period. The annual plots for each organization are combined in a single plot to provide insights into the data that is disseminated to all participating organizations.

The CESR DCC has developed a set of SAS macros for both distributed QA and centralized QA. For the distributed QA, the SAS macro programs are sent to the organizations in the QA package. These macros run on datasets and input variables to create data summaries described above. They include standardizing the QA checks for reporting; issuing warning, fail, and pass messages; hiding low counts according to standards set by each organization; and creating a summary data file of the results. The process of combining QA meta-file results from each organization into one single data structure is also done using a macro that helps efficiently create and update the multi-organization reports. A detailed discussion of organizational level QA checks are out of scope for this paper, and, instead, we focus on the data knowledge gained from combining the results.

### CESR VDW Quality Assessment Reports

Two levels of reporting are performed for QA of each content area:

1. The single organization QA report includes all the QA check results and the detailed results of all checks and outcomes including passes, warnings, and fails that make it easy for data managers to quickly identify the issue that requires correction and understand the quality of the single table at the time of the QA.
2. The multi-organization report is created when individual organizations share the DQA summaries with the DCC.

The findings from the multi-organization quality assessment are communicated to data partners through individual person-to-person communications and group presentations to all the data mangers, programmers, and data stewards. These meetings facilitate conversations and allow users to share insights into the data. The minutes from the presentation and the subsequent discussions are also communicated to all data managers. The final reports are saved in a centralized portal for access by all KP data managers, investigators, and DCC personnel. The multi-organization reports are comprehensive, extensively discussed amongst DCC and organization data managers, and uploaded at the end of each QA process for use by all and are often a reference for specific research related questions.

## Results

Our experience has taught us that collecting data that is generated outside research work is complex because we lack *a priori* expectations and methods to examine these data. We also do not fully understand the context of data collection and this prevents us from understanding the expected values of data elements, their completeness, and expected direction of change in the future.

In the absence or inapplicability of published standards to the population, combining data summaries from different organizations allow expectations about the data elements. For instance, when we plot results from all organizations together in the multi-organization reports, it is easy to identify trends, outliers, gaps, or spikes. Considering the outcomes of several checks simultaneously reveals insights on data availability, collection methods, differences in utilization due to differing health plans or programs being in place.

In this section, we show how by comparing annual trends of two data content areas—social history and death—we can obtain insights into the data quality. The VDW social history data has a single data source in more recent years which is the vendor provided EHR (i.e., EPIC-Clarity EHR), even though the set-up of the EHR is somewhat different between organizations. In contrast, the death table has multiple data sources that are internal and external to the organizations.

### Known EHR patterns seen in the VDW

At the organizations involved in KP CESR VDW, social history information is captured in the outpatient clinic and is usually patient reported. Inquiries into the data generating processes reveal that every time a patient completes social history questions, a new record is created in the system regardless of there being any changes in the person’s social history. In addition, the amount of data collected for each individual might vary. For example, a self-reported smoker might get more questions about his smoking status than a person who reports ‘never’ for smoking status.

**Figure 2:**
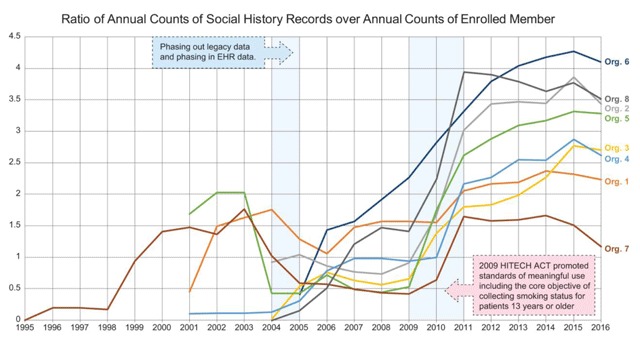
Ratio of Annual Counts of Social History Records over Counts of Enrolled Members per Year.

Figure 2 illustrates a few key trends in data collection within and across organizations. The plots are the ratio of total records in the social history table captured per year over the total number of enrolled members in the organization. The EHR data started in 2004 for all KP organizations. Prior to 2004, four out of the eight organizations had legacy data that were added to the VDW social history table. The drop in the record counts from 2004 to 2010 is not a gap in the data but a result of a move from legacy data systems to the EHR and the gradual expansion to the EHR thereafter. In 2010, additional data elements were added that led to increase in record counts per member across all organizations. This was a direct result of the Health Information Technology for Economic and Clinical Health (HITECH) Act, part of the American Recovery and Reinvestment Act (ARRA) of 2009. The HITECH Act promoted standards of meaningful use including the core objective of collecting smoking status for patients 13 years or older.

Some of the differences in the trend lines could be attributed to the differences in the organizational structure. For example, Org. 7 has fewer owned- hospitals and clinics and capture more claims data than other sites. Hence the record counts in social history, which is encounter-specific, is lower than most of the other sites, while still having a higher enrollment count.

The multi-organization comparison goes beyond single site analysis by confirming that the steep rise in records is not anomalous for a single organization, but a common occurrence across organizations. The implication of these findings is that the variations in the social history items are likely to reflect true variations in the clinical methods and/or member population across organizations from 2011 onwards. Projects requiring social history data can expect to obtain reliable data from all organizations from 2011 to present [3].

### Difference in source data

Unlike for social history, the source of death data varies across organizations, which represents a real challenge to combining data from different sources. For each patient in the death file, there are one or more data sources indicating that the patient is deceased. However, a data source may indicate death without a specific death date. Additionally, when death dates are present, there are often inconsistencies in the data, such as enrollment continuing after death or encounters, blood pressure measurements, or prescription dispenses are recorded after the death date. A small number of these cases are possible within reasonable boundaries (<0.5 percent of all records) due to health plan system processes needing time to close out. All site data managers should ensure that this is corrected within the EHR system for subsequent data pulls.

**Figure 3:**
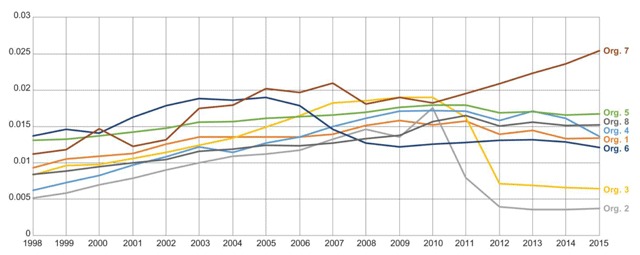
Ratio of Annual Counts of Death Records over Counts of Enrolled Members per Year.

Figure 3 shows the ratio of the annual counts of death records of all members (regardless of their enrollment status) over annual counts of enrolled members. Although four of the eight organizations show similar trend from 2010 onwards, there is significant variation amongst the remaining organizations.

Most organizations are dependent on Social Security Administration or state/national death index data as events outside the organization’s health plan may not be captured in the EHR. While these sources provide vital status information on members who might not be enrolled any longer, there are delays in obtaining the data as we see for Organizations 2 and 3. Thus, death data may be incomplete in recent years.

**Figure 4:**
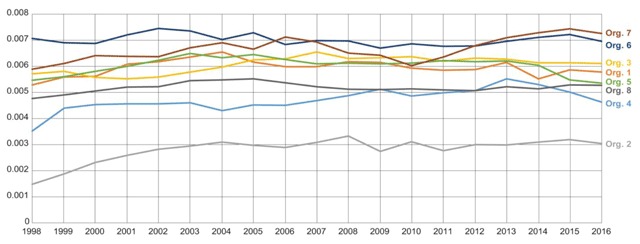
Ratio of Annual Counts of Death Records (for enrolled members only at the time of death) over Counts of Enrolled Members per Year.

We provide an additional figure from the DQA that should also be considered when assessing data completeness of the death data. Figure 4 shows the death counts of deceased members who were enrolled during the time of death. The trend line in this figure is stable for all organizations, including Organizations 2 and 3, over time. This implies that the algorithms at all organizations are consistently capturing death records for enrolled members.

The death rates for organizations 2 and 4 are lower because neither of these organizations capture death data from their state or national data sources. These two sites likely don’t have complete capture of death information for patients who died outside of their local health plan facilities.

## Discussion

The CESR DCC’s methodology for DQA and reporting has several beneficial externalities that could be used by researchers participating in multi-site research. To create the DQA methodology, the CESR DCC developed codes and procedures for efficiently implementing and reporting the DQA across multiple organizations. The CESR DCC approach is to:

1. distribute DQA tools so that data managers at each organization are empowered to assess data at any time,
2. disseminate findings from DQA to improve overall data quality, and
3. engage all data managers and end-users in an exchange of knowledge about the data.

The single and multi-organization reporting fulfills the dual purpose of organizational independence as well as organizational coordination and interdependence. As there are growing costs of managing data in any single organization, collaborations on data management will not only decrease the cost, but also increase the quality of complex data systems.

Multiorganization reporting provides additional insight on data quality by validating trends across organizations. There are several feedback loops between the DCC and the individual data managers during a DQA process that inform both the organization and the DCC on the data quality. The individual organization’s data managers provide data quality assurance at the data warehouse level with feedback from the DCC DQA process. The regularity with which the DCC conducts and reports DQA builds data user trust, prevents extra work and time, and allows for easy replication of research work.

In health care research, analytical techniques and corresponding data requirements are often developed in isolation due to the project funds being awarded to a group/team led by a few research investigators. In this scenario, a centralized platform like the DCC avoids “organizational silos” by bringing together disparate teams and organizations with the purpose of improving overall data quality of distributed databases, understanding the processes that lead to the creation of the data, and assessing data for quality. As seen from the examples above, the CESR DCC DQA process can identify common trends in record counts (social history) as well as differences in record counts due to multiple data sources (death) for each organization. This can inform optimal budget allocations between various activities including obtaining additional data needs, cleaning the data for project specific needs or developing analytical techniques dependent on availability of reliable data. The consistent and timely multiorganizational DQA reporting is a part of pre-research data review that allows combining multi-organization data for a single research project.

The DQA is completed for all records in the data for one content area. The datasets are assessed for subgroups like social history record counts for male versus female, or total record counts by year, etc. The DQA checks are never completed on any single disease group or clinical procedures to inform a specific project. This is because DQA of the VDW is intended to support a multitude of projects and should not be a substitute for project-specific quality assessment. CESR DCC makes the DQA programs and methods available to researcher projects who are interested in using them for project specific assessments. In addition, the CESR DCC provides expertise on specific data content. At the individual organizational level, data user feedback is incorporated by organizational data managers and applicable knowledge is transferred to other data managers through CESR meetings.

With datasets increasing in size, performing DQA on complete set of data for larger tables that include multiple records on almost all members or multiple records for each encounter is becoming more difficult. For example, checking the linkage or patient-encounter identification between social history encounters to other encounter-based tables would not be feasible due to the size of the utilization tables. One way to address this problem is by testing the linkage of a 10 percent random sample. Another way is to test for linkage of encounters to social history by selecting only the past six months’ worth of data for both tables. As the data sources become more complex, more efficient programming techniques or increased computer capacity are required to assess the data quality.

### Implications for clinical research data networks

The multi-organization quality assessment helps multi-organization research. DQA reports (for example, category or range values by age-group) provide a quick overview of data quality across organizations to determine which organizations have the necessary population (patient or disease) to qualify for research and be included in a research proposal. The purpose of multi-organization comparisons, either at the level of data warehouse maintenance or project-based data cleaning efforts, is twofold. First, they reduce variabilities and errors in data capture, so that what remains is variability either due to inter-regional differences in the health care administration or the true variation in the data that would be helpful towards a comparative effectiveness study. Second, they document differences to allow for appropriate data inclusion and processing. The funding opportunities also improve across regions with trust and confidence in the data quality.

## Conclusions

This paper described a multi-organization reporting process as an advancement of the DQA process that the CESR DCC has implemented since 2009. A centralized DCC improves network knowledge and informs inter-organizational research that are by-products of a good DQA process. By continued multi-organization reporting not only does the DCC provide services to data managers across all organizations, but it also creates an easily accessible repository of the knowledge gained. These procedures can help any researcher conducting multi-organization research where data quality is a paramount concern.

## Footnotes

CESR DCC has developed and distributed a separate QA package for assessing data refresh. The CESR Refresh QA package is available upon request but further discussion on the QA that aligns with refresh cycle is out of scope for this paper.
